# A Standardized Collagen-Based Scaffold Improves Human Hepatocyte Shipment and Allows Metabolic Studies over 10 Days

**DOI:** 10.3390/bioengineering5040086

**Published:** 2018-10-16

**Authors:** Marc Ruoß, Victor Häussling, Frank Schügner, Leon H. H. Olde Damink, Serene M. L. Lee, Liming Ge, Sabrina Ehnert, Andreas K. Nussler

**Affiliations:** 1 Department of Traumatology, Siegfried Weller Institute, Eberhard Karls University, 72076 Tübingen, Germany; m.ruoss@hotmail.de (M.R.); victor.haeussling@hotmail.de (V.H.); sabrina.ehnert@gmail.com (S.E.); 2 Matricel GmbH, 52134 Herzogenrath, Germany; schuegner@matricel.de (F.S.); olde_damink@matricel.de (L.H.H.O.D.); 3 Hepacult GmbH, 82152 Martinsried/Planegg, Germany; Serene.Lee@med.uni-muenchen.de (S.M.L.L.); liming.ge@hepacult.de (L.G.); 4 Biobank of the Department of General, Visceral and Transplantation Surgery, Hospital of the LMU, 81377 Munich, Germany

**Keywords:** drug-induced hepatotoxicity, pre-clinical drug testing, cells shipment, natural collagen scaffolds

## Abstract

Due to pronounced species differences, hepatotoxicity of new drugs often cannot be detected in animal studies. Alternatively, human hepatocytes could be used, but there are some limitations. The cells are not always available on demand or in sufficient amounts, so far there has been only limited success to allow the transport of freshly isolated hepatocytes without massive loss of function or their cultivation for a long time. Since it is well accepted that the cultivation of hepatocytes in 3D is related to an improved function, we here tested the Optimaix-3D Scaffold from Matricel for the transport and cultivation of hepatocytes. After characterization of the scaffold, we shipped cells on the scaffold and/or cultivated them over 10 days. With the evaluation of hepatocyte functions such as urea production, albumin synthesis, and CYP activity, we showed that the metabolic activity of the cells on the scaffold remained nearly constant over the culture time whereas a significant decrease in metabolic activity occurred in 2D cultures. In addition, we demonstrated that significantly fewer cells were lost during transport. In summary, the collagen-based scaffold allows the transport and cultivation of hepatocytes without loss of function over 10 days.

## 1. Introduction

Drug-induced hepatotoxicity is the leading cause of acute liver failure and post-marketing withdrawal of drugs. Thus, a major challenge when developing new drugs is their assessment of undesired side effects in humans [1]. In order to ensure the highest degree of safety and efficacy of a potential drug, many time-consuming and expensive pre-clinical tests must be performed. Consequently, to limit developmental costs there is an enormous demand for predictive in vitro test systems in order to discriminate, as early as possible, between promising and inadequate drug candidates. Pre-clinical drug testing is typically done using animal models; however, animal models used by the pharmaceutical industry are, so far, not very representative for the human situation [2,3,4]. Because of the poor predictive power of current preclinical models, 90% of the drugs approved for the clinical phase do not enter the market or must be taken off the market on the basis of long-term experience in humans [5]. The development of an in vitro test system that allows the early detection of hepatotoxicity would not only reduce time and costs for drug testing but also increase human safety and reduce animal tests.

It is widely accepted that reliable predictions could only be made with a human in vitro models to exclude or further develop new drugs [6]. However, all of these models show several limitations. So far, many different cell types including (fresh, cryopreserved, and immortalized) primary hepatocytes [7,8,9], hepatic cell lines [10], and stem cell-derived hepatocyte-like cells [11] have been tested. However, only freshly isolated primary human hepatocytes (PHHs) show a metabolic profile that is comparable to the in vivo situation [12], making them the currently used ‘gold standard’. However, usage of PHHs has also its disadvantages.

Firstly, the origin of these cells is mostly from already medicated tumor patients. Therefore, the cells could have an altered metabolism, which does not represent the situation in the disease the drug is designed for. Furthermore, the dependence on human donors implies that the cells are not available in sufficient quantities anytime and anywhere [13].

Secondly, cryopreservation of the cells is also a limited alternative, because it is frequently associated with an enormous loss in number and function of the PHHs [14]. Therefore, PHHs are mostly shipped as a cooled suspension on ice [15]. However, this kind of transport also damages the cells by hypothermia. Thus, the viability of the PHHs decreases during transport and the remaining cells show impaired cell attachment and reduced function [16]. In general, transportation of PHHs at 37 °C, where cells remain largely intact, is possible but this form of transport is logistically unwieldy: (i) before cells can be shipped it is mandatory to wait until cells attach onto the culture plastic; (ii) plates have to be sealed tightly and packages have to be handled carefully not to spill the medium, as loss of medium (dry wells) stresses the cells and increases the risk for contaminations; and (iii) additionally, plated cells require much more space compared to the transport in suspension.

Last but not least, hepatocytes lose their metabolic activity in the conventional 2D culture after a few days [15,17]. Therefore, in recent years, an increasing number of approaches have been focused on the cultivation of hepatocytes in 3D culture systems, as a 3D environment is supposed to improve the metabolic function of the cells. In these systems, different matrices, either derived from natural or synthetic compounds, were used to mimic a 3D environment. From these studies it is known, that an ideal scaffold should have an interconnected pore structure and a high porosity to ensure penetration of the cells, as well as adequate diffusion of nutrients to cells and of waste products away from the cells within the scaffold [18].

Cells interact with scaffolds mainly via chemical groups (ligands) on the material surface. Scaffolds synthesized from natural extracellular materials (e.g., collagen) naturally have these ligands in the form of Arg-Gly-Asp (RGD) binding sequences. The ligand density is determined by the specific surface area (within a pore) to which cells may adhere. Thus, the mean pore size of a scaffold represents another key component defining cell attachment, survival, and function [19,20]. In addition to the pore size, porosity and the water-uptake capacity of the scaffold are also important parameters in cell-scaffold interaction. They determine the interconnectivity of the pores, how deep the cells can penetrate into the scaffolds and whether the cells inside the scaffold can be supplied with nutrients [21]. Besides, the stiffness of the surface also has a huge influence on the functionality of the cells [22]. Especially in liver the stiffness is often associated with different disease states. While a healthy liver has a stiffness of approximately 6 kPa, a fibrotic and cirrhotic liver is much stiffer (>12.5 kPa to 75 kPa) [23].

Besides using 3D matrices, fluid-flow systems were also established to preserve the metabolic function of PHHs over a long time period [15]. Although these attempts were able to maintain the metabolic activity of the cells a bit longer than in 2D cultures, up until now all of these recent 3D scaffolds had limitations [18]. The increasing complexity of a model in combination with the high variance of human material makes standardization almost impossible. Additionally, these scaffolds are often not suitable for 96-well plates. Thus, these systems can barely be used for high-throughput methods. In addition, these systems have been explored so far mainly with rat hepatocytes instead of human ones [24,25,26], which makes the translation extremely difficult for testing of new drugs.

Thus, the aim of this study was to test the well standardized Optimaix-3D Scaffold, which can be used for several standard plate formats, including 96-well plates, for its suitability for PHHs transport and long-term functional culture. The physical characterization of the novel collagen-based scaffold includes the determination of its pore size, porosity, permeability, water-uptake capacity, and stiffness. It was further tested whether the 3D cultivation allows the cells to be sent more gently and to be cultivated over 10 days without loss of viability. In addition, the influence of the scaffold cultivation on the main metabolic functions (CYP activities, urea, and albumin synthesis) of PHHs was measured for up to 10 days and compared to conventional 2D cultures.

## 2. Materials and Methods

### 2.1. Scaffold Manufacturing

The collagen scaffolds (Optimaix-3D) used in these studies were produced by the company Matricel. The scaffold manufacturing process is based on a so-called directional solidification method [27]. In brief, the developed method for scaffold manufacturing starts with the preparation of a homogeneous aqueous dispersion of collagen. In the subsequent controlled freezing process, finger-shaped ice crystals that grow through the dispersion are generated, so that the collagen fibers are not trapped within the ice crystals but concentrate in the interstitial space. During the subsequent freeze-drying, the ice sublimes and the open porous collagen structure remains. This basic collagen scaffold structure is further cross-linked with 1-ethyl-3-(3-dimethylaminopropyl) carbodiimide hydrochloride (EDC) in order to adapt the stability against degradation by cells that are cultivated within the scaffold.

### 2.2. Physical Characterization of Scaffolds

#### 2.2.1. Pore Size

To analyze the pore size, the organic matrix components of the scaffold were stained with sulforhodamine B (0.08% SRB in 1% acetic acid), which binds to protonated amino acids under acidic conditions. Unbound SRB was removed by washing the scaffolds three times with 1% acetic acid solution. With the red fluorescent signal of the bound SRB, the porous structure of the cryogels could be visualized with a fluorescence microscope (EVOS FL AF 4301, life technologies, Darmstadt, Germany). Using the ImageJ software, version 1.5 (National Institutes of Health, Bethesda, MD, USA), the shape and size of the pores were determined [21].

#### 2.2.2. Porosity

The porosity of the scaffold was calculated using a method published by Shimizu et al. [28]. The porosity in percent was determined using the equation:

$$\mathrm{Porosity}\left(\%\right)=(1-\left(\frac{\left(\text{scaffold wet weight}\left(g\right)-\text{scaffold dry weight}\left(g\right)\right)}{\left(\text{scaffold volume}\left({\mathrm{mm}}^{3}\right)/\text{density of water}\left(g/{\mathrm{mm}}^{3}\right)\right)}\right)\times 100\%,$$

The volume of the scaffolds was calculated based on its diameter (5 mm) and height (1.5 mm) to be 29.5 mm^3^. The dry weight of the scaffolds in grams was determined with an analytical balance. Scaffolds were then submerged in sterile water for 1 h and were weighed to obtain the wet weight of the scaffolds in grams.

#### 2.2.3. Permeability

The permeability of the scaffold was calculated by a method published recently [29]. Briefly, a stable hydrostatic pressure was applied to the top surface of the porous scaffold. The quantity of water permeated through the scaffold per minute was weighed and then used to calculate the permeability according to Darcy’s law.

$$\mathrm{Porosity}\left(\mu m^{2}\right)=\frac{\text{viscosity water}\left(\mathrm{Pa}\times s\right)\times \frac{\text{water passed through the scaffold}{\mathrm{mm}}^{3}}{\min}}{\frac{\text{cross sectional area of the scaffold}\;\left({\mathrm{mm}}^{2}\right)\times \text{constant pressure}\;\left(\mathrm{Pa}\right)}{\text{length of the scaffold}\;\left(\mathrm{mm}\right)}},$$

To determine the volume of water that passed through the scaffold per minute, the water was collected and weighed (g) using an analytical balance. Multiplication with the specific density of water (0.997 g/cm^3^) gave the required volume in mm^3^.

To determine the constant pressure applied, the height of the water column (90.1 mm) was multiplied by the cross-sectional area of the scaffold (78.5 mm^2^). The weight of the resulting water volume (in average 2.5 g/s) was calculated to be 150 cm^3^/min by multiplying the water volume with the specific density of water (0.997 g/cm^3^). By dividing with the gravitational force (~9.8 m/s^2^) the constant pressure applied was determined to be 881 Pa.

#### 2.2.4. Water-Uptake and Swelling Ratio

The water-uptake and swelling ratio were obtained according to a previously described method [30]. The water-uptake as well as the swelling ratio was calculated according to the following equations:

$$\text{Swelling ratio}\left(\%\right)=\frac{\left(\text{scaffold wet weight}\left(g\right)-\text{scaffold dry weight}\left(g\right)\right)}{\text{scaffold dry weight}\left(g\right)\times 100},$$

$$\text{Water uptake}\left(\%\right)=\frac{\left(\text{scaffold wet weight}\left(g\right)-\text{scaffold dry weight}\left(g\right)\right)}{\text{scaffold wet weight}\left(g\right)\times 100},$$

The dry and wet weights (in grams) of the scaffolds were determined with an analytical balance as described above.

#### 2.2.5. Matrix Stiffness

The scaffold stiffness is defined by the Young’s modulus, which describes the ratio of stress *σ* and strain *ε* independent of the size or the shape of samples. Briefly, Optimaix-3D scaffolds (3 mm height × 10 mm diameter) are compressed four times uniaxial, by a cyclic compression of 10% height, with a velocity of 5 mm/min, using a ZwickiLine Z 2.5TN (Zwick GmbH & Co. KG, Ulm, Germany). The required load is measured real-time by a Xforce HP 5N sensor (Zwick GmbH & Co. KG (Ulm, Germany) The resulting load-deformation curve is converted into a stress-strain curve, using the area and initial sample height. In the region of linear elastic deformation, the Young’s modulus [31] is then calculated, using the following formula:

$${\mathrm{Young}}^{\prime }s\;\text{modulus}\left(\mathrm{MPa}\right)=\frac{\text{applied force}\left(N\right)\times \text{initial scaffold height}\left(\mathrm{mm}\right)}{\text{area of the scaffold}\left({\mathrm{mm}}^{2}\right)\times \text{change in height}\left(\mathrm{mm}\right)},$$

### 2.3. Hepatocyte Isolation, Shipment, and Culture

PHHs were isolated from liver resections by a two-step EDTA/collagenase perfusion technique as described previously [32,33]. Double-coded liver pieces used for PHHs isolation were provided by the Biobank of the Department of General, Visceral and Transplantation Surgery in Ludwig-Maximilians University (LMU). This Biobank operates under the administration of the Human Tissue and Cell Research (HTCR) Foundation. The framework of HTCR Foundation [34], which includes obtaining of written informed consent from all donors, has been approved by the ethics committee of the Faculty of Medicine at the LMU (approval number 025-12) as well as the Bavarian State Medical Association (approval number 11142) in Germany.

PHHs were shipped overnight as a cell suspension with up to a maximum of 5 × 10^7^ viable cells per mL Cold Storage Solution (Hepacult GmbH, Martinsried/Planegg, Germany), in cryo-vials on ice [32].

Upon arrival, cells were washed once with PBS. The cell number and viability was determined by Trypan blue exclusion method, using a Neubauer counting chamber. To improve the viability, a Percoll density gradient centrifugation was performed for 20 min at 1300 g (Percoll solution was diluted with PBS to obtain a total density of 1.0675 g/L). The PHHs were washed once with PBS and resuspended in Williams Medium E supplemented with 10% fetal bovine serum, 100 U/mL penicillin, 0.1 mg/mL streptomycin, 15 mM HEPES (pH 7.0–7.6), 1 mM Glutamine, 1 mM sodium pyruvate, 1 mM human insulin, 0.8 μg/mL hydrocortisone and 1% nonessential amino acids. Cells were counted again and seeded onto culture dishes or the Optimaix-3D scaffolds from Matricel.

**Figure 1:**
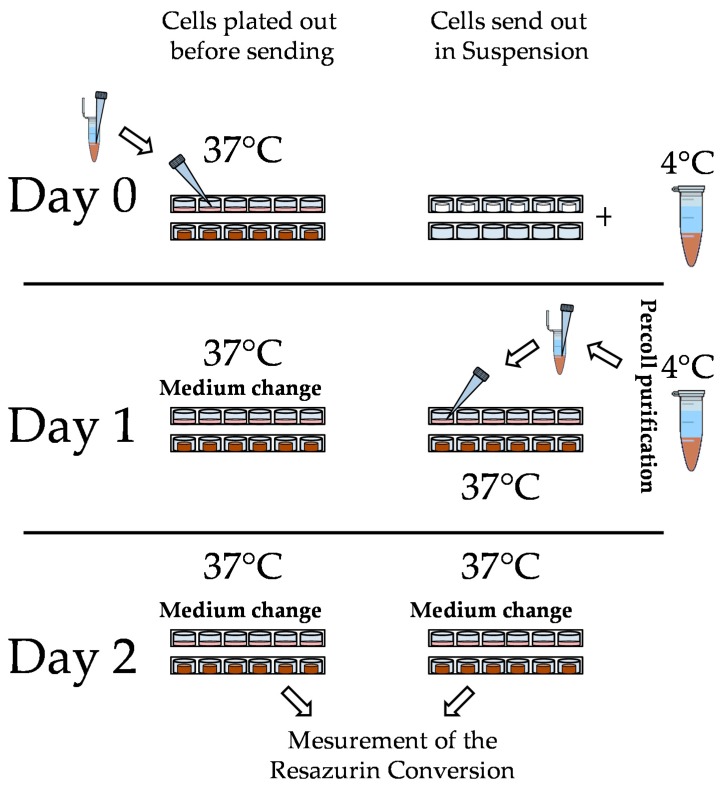
Schematic illustration of the transport schemes: on day 1, the primary human hepatocytes (PHHs) were purified and sent either in suspension or directly plated onto 2D culture plates and Optimaix-3D scaffold. Then, cells were sent at 37 °C (plated cells) or at 4 °C (cells in suspension) overnight. On day 1, the medium was changed in the plated cultures, while the cells in suspension were purified and plated out (2D, Optimaix-3D scaffold). Two days after isolation, a viability determination of all conditions was carried out by measuring resazurin conversion.

Both in 2D and 3D 0.3 × 10^6^ million cells were plated. For better cell adherence 24-well culture dishes that were used for 2D culture were coated with rat tail collagen as described [9]. For cell seeding on the scaffold, the so called ‘Drop-on’ seeding method, recommended by the manufacturer, was used [35]. Therefore, cells were re-suspended in the medium at a concentration of 10 million PHHs/mL. 30 μL of this cell suspension was pipetted onto the scaffold to achieve nearly complete rehydration. After an attachment period of 2 h, additional medium was added (500 μL/well, 24-well-plate) to the scaffolds. The scaffolds fit also into a single well of a 96-well plate; however, in order to increase the comparability between 2D and 3D (same amount of medium), 24-well plates were also used for metabolic activity measurements of the 3D culture. To test whether the scaffold was also suitable for the shipping of the PHHs, cells were plated after isolation as described above in 2D and onto Optimaix-3D scaffolds. The exact procedure is schematically shown in Figure 1. Both cultures as well as cells in suspension were sent at 37 °C overnight. At the next day cells were purified as descripted before and plated out in 2D or onto Optimaix-3D scaffolds at the same concentration as described before. For comparison of the viability of each condition a measurement of resazurin conversion was carried out as described in Section 2.4.4.

### 2.4. Functional Testing

Main metabolic functions, like urea and albumin production and activities of CYP enzymes were measured on days 3 and 10 of culture. For normalization, Resazurin conversion was determined.

#### 2.4.1. Urea Measurement

The cells were washed once with PBS. Subsequently, the urea quantification was carried out, using a protocol described by Seeliger et al. [11]. Briefly, cells were incubated for 24 h with medium without additives, in the presence or absence of 5 mM NH_4_Cl or 5 mM NH_4_Cl and 0.1 M ornithine. 80 μL of the supernatant was mixed with 60 μL of *O*-phthalaldehyde solution (1.5 mM *O*-phthalaldehyde, 4 mM Brij-35, 0.75 M H_2_SO_4_) and 60 μL of NED reagent (2.3 mM *N*-(1 Naphthyl) ethylenediamine dihydrochloride, 0.08 M boric acid, 4 mM Brij-35, 2.25 M H_2_SO_4_) and incubated for 1 h at 37 °C. The absorbance was measured at 460 nm and compared to a urea standard curve (0–100 μg/mL) on the same plate.

#### 2.4.2. Albumin ELISA

The produced albumin was quantified with the human albumin ELISA kit (E80-129 from Bethyl Laboratories, Montgomery, USA) according to the manufacturer’s instructions. Briefly, 96-well-plates were coated with the primary antibody for 1 h at room temperature (RT). After washing five times with washing buffer (50 mM Tris, 140 mM NaCl, 0.05% Tween 20), unspecific binding sites were blocked with blocking solution (50 mM Tris, 140 mM NaCl, 1% BSA) for 1 h at RT. After another 5 washes, albumin standard and sample (diluted 1:50 in sample conjugate buffer) were applied to the plate and incubated for one hour at RT. After another 5 washes, the secondary antibody was added to each well and incubated for 1 h at RT. After the last 5 washes, the luminescence solution (100 mM Tris, 125 nM Luminol, 200 nM p-Coumaric acid, 0.08% 30% H_2_O_2_ solution) was pipetted into the wells and the luminescence was measured in the Omega plate reader BMG LABTECH, Ortenberg, Germany. The albumin quantity was calculated using a standard curve.

#### 2.4.3. CYP Activity Measurement

CYP enzyme activities of CYP2B6, CYP2D6, CYP2C9 and CYP3A4, being responsible for the metabolism of most drugs [36], were measured as recently described [37]. Briefly, the chosen substrates, the selected concentrations, the incubation times and the measured metabolites are summarized in Table 1. Methanol, which was the initial solvent of the CYP substrates, was removed before use by evaporation, and the substrates were dissolved in culture medium. The cells were incubated with 500 μL of the respective reaction solution. After the described incubation times, the supernatants were removed and frozen at −80 °C until measurement. The enzymatic activity was measured by the company Pharmacelsus (Saarbrücken, Germany) using a LC-HPLC/MS-based methodology [37].

#### 2.4.4. Resazurin Conversion

After each functional test, the wells/scaffolds were washed once with PBS and then incubated with a 0.0025% resazurin solution (in medium) for 1 h at 37 °C. The fluorescence of the produced resorufin was measured at 544 nm/590-10 nm using the Omega Plate Reader [38].

#### 2.4.5. Statistic

Statistical significance of differences between two groups was evaluated by non-parametric Mann-Whitney-U-test. For comparison of the differences between more than two groups, non-parametric Kruskal-Wallis H-test followed by Dunn’s multiple comparison test was performed (GraphPad Prism 5.00 Software, San Diego, CA, USA). Data are represented as means  ±  SEM of at least three independent experiments (N ≥ 3). All statistical comparisons were performed two-sided in the sense of an exploratory data analysis using *p* < 0.05 (*), *p* < 0.01 (**), and *p* < 0.001 (***) as level of significance.

## 3. Results

### 3.1. Characterization of the Optimaix-3D Collagen Scaffold

Optimaix-3D scaffolds were prepared by a so-called directional solidification method, which includes subsequent freeze-drying cycles [27]. Scaffold properties are summarized in Table 2.

**Figure 2:**
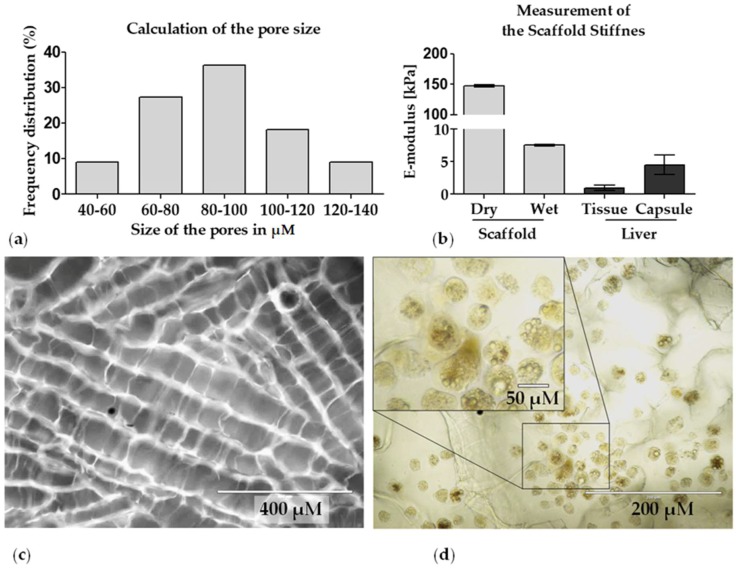
Physical characteristics of the Optimaix-3D scaffold: (**a**) pore size distribution was determined with the ImageJ software. (N = 4, *n* = 5). (**b**) The stiffness of the scaffold was measured with the help of a Zwick material testing machine (N = 4, *n* = 3). (**c**) Representative fluorescent microscopic picture of the pore structure in the Optimaix-3D scaffold. For visualization Optimaix-3D scaffolds were stained with SRB. (**d**) Microscopic image of the scaffold colonized with PHHs.

The pore size (mean diameter) of the scaffold, which was measured from four independent scaffolds with the ImageJ software (five pores/scaffold were analyzed), ranged from 55–140 μm, with the most pores being between 80 μm and 100 μm (Figure 2a,c). The porosity of the scaffold that were measured four times independently was approximately 96%. Together with the high permeability of 54 μm^2^, which was measured from three independent scaffolds (three times each scaffold), this is an indicator for a high interconnectivity of the pores [39]. This high porosity even allows visual analysis (light microscopy) of the plated PHHs. Normally, in a scaffold culture, it is not possible to take light micrographs, but the high porosity of the Optimaix-3D scaffolds (height = 1.5 mm) allows enough light to penetrate the scaffold for light microscopy (Figure 2d). The water-uptake of the Optimaix-3D scaffold was >97%. Together with the swelling ratio of 3387%, this represents the strong hydrophilic nature of the scaffold. The dry Optimaix-3D scaffold has a high stiffness of 148 kPa, which allows easy handling of the scaffolds. When soaked with medium, the wet Optimaix-3D scaffold showed a stiffness of nearly 7.5 kPa (Figure 2b) which is comparable to the stiffness of the healthy human liver [23].

### 3.2. Loss of PHHs During Transport Is Significantly Reduced When Shipped on Optimaix-3D Scaffold

**Figure 3:**
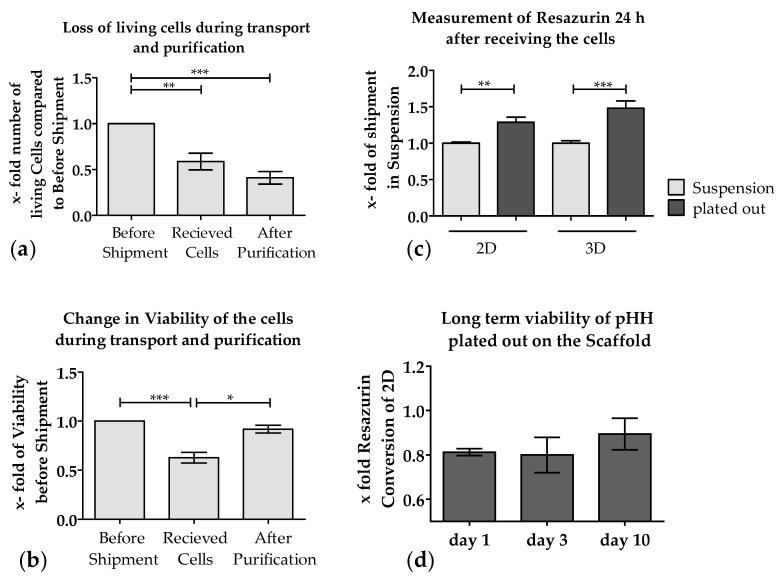
Effect of shipment on the viability and adherence of PHHs. Freshly isolated PHHs were shipped overnight in cold storage solution. Total cell numbers and the viability of the cells were measured before shipping, before and after Percoll purification. (**a**) Amount of living PHHs decreased during transport and purification. (**b**) Viability of PHHs can be increased by using Percoll density purification. (**c**) Viability (Resazurin conversion) of PHHs shipped overnight either adherent or in suspension (with purification and plating upon arrival). Data shown represent an average of N = 9 (PHH were shipped and purified nine times independently (**a**,**b**). (**c**) (N = 3, *n* = 20) cells were shipped three times independently, cultured both directly on scaffold and onto plates (2D) or in parallel in suspension and then plated after shipment. (**d**) shows long time viability of PHH plated out on scaffold over 10 days that were quantified by measurement of the resazurin conversion (values of 3D were normalized to the values of 2D on the same day N = 3, *n* = 3 (mean ± SEM). * *p* ≤ 0.05; ** *p* ≤ 0.01; *** *p* ≤ 0.01).

So far, it has not been possible to send PHHs on ice or cryopreserved without a massive loss of function and viability [40,41]. Therefore, we investigated if it is possible to ship PHHs plated onto Optimaix-3D scaffolds (Matricel) compared to conventional 2D culture plates overnight. Nowadays nearly half of the PHHs shipped in suspension in cold storage solution get lost during transport [41], as can be seen in Figure 3a, which include data of nine shipments. An actual comparison that includes data of the 2D/3D shipment experiments can be found in Table 3. This was partly due to the decreased viability of the cells upon arrival (Figure 3b), which raised the need for additional purification step, as the proteases released from the dead PHHs may damage the healthy cells and adversely affect their adherence. Thus, for purification, a Percoll density centrifugation was typically performed, which resulted in increased viability of the cells (Figure 3b) but decreased significantly the number of viable cells (Figure 3a).

Although the loss in viability resulting from shipment was compensated by the Percoll density purification, the cells that were sent in suspension and subsequently plated showed significantly lower adherence compared to cells directly cultured onto Optimaix-3D scaffolds or on collagen-coated plates (2D) (Figure 3c). When comparing PHHs sent in 2D with those on the Optimaix-3D scaffolds, it is remarkable that the cells on the Optimaix-3D scaffolds survive the shipping better than the cells in 2D-culture (Figure 3c). As shown in Figure 3d, approximately 80% of the cells adhere on the scaffold surface compared to 2D, which can be explained by the fact that some cells are rinsed off after 2 h when medium is added. However, the relative viability on the scaffold is close to the cells cultured in 2D.

### 3.3. Metabolic Function of PHHs on the Optimaix-3D Collagen Scaffold

The next step of the present study was to monitor the hepatic function of the PHHs cultured on this new scaffold in comparison to the conventional 2D culture. It is well recognized that cultured PHHs rapidly lose their metabolic function in vitro [12]. Therefore, to investigate if PHHs cultured on Optimaix-3D scaffolds maintain their metabolic function longer than in 2D cultures, we analyzed the main hepatic functions like CYP activity, urea, and albumin synthesis on days 3 and 10 of culture.

#### 3.3.1. 3D Environment by the Optimaix-3D Scaffolds Supports Urea Production in PHHs

**Figure 4:**
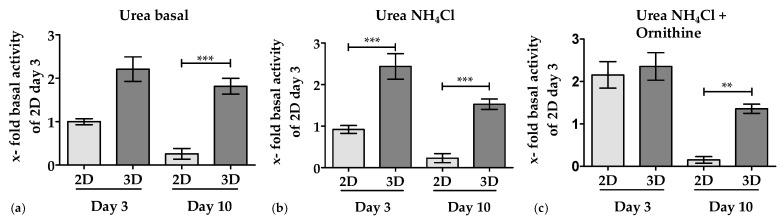
Urea production and ammonia detoxification. (**a**) Basic urea production by PHHs over 24 h in 2D (bright bars) and 3D (dark bars) cultures. (**b**) Urea production by PHHs after 24 h following the incubation with NH_4_Cl in 2D (bright bars) and 3D (dark bars) cultures. (**c**) Urea production by PHHs after 24 h incubation with NH_4_Cl plus ornithine in 2D (bright bars) and 3D (dark bars) cultures. Data were normalized to the resazurin conversion (viable cells). Data represent an average of N = 3 independent experiments (*n* = 3). Bars represent mean ± SEM ** *p*≤ 0.01; *** *p* ≤ 0.001 as indicated.

The basal urea production of PHHs cultured on Optimaix-3D scaffolds was approximately 50% higher compared to PHHs in 2D cultures. Furthermore, basic urea production of PHHs in 2D cultures rapidly declined within 10 days. In contrast, the basic urea production in 3D cultures remained constant over 10 days (Figure 4a). Upon addition of ammonia, cells in a 3D culture produced significantly more urea on day 3 and day 10 than in 2D cultures, clearly demonstrating an improved detoxification capacity in 3D cultures (Figure 4b). In 2D cultures, however, this could only be obtained on day 3, when cultures were supplemented with the co-factor ornithine (Figure 4c).

#### 3.3.2. 3D Environment of the Optimaix-3D Scaffolds Favors Albumin Synthesis in PHHs

**Figure 5:**
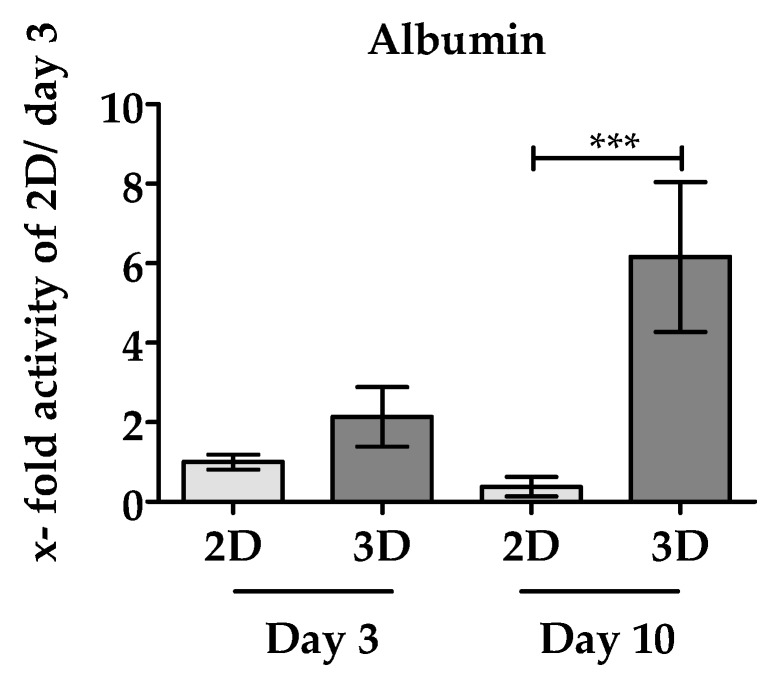
Synthesis of albumin by PHHs in 2D and 3D culture. Supernatants after 24 h incubation time were collected on day 3 and 10 of culture. Albumin production was measured by human Albumin ELISA. Results of 2D cultures are given as bright bars, results of Optimaix-3D cultures are given in dark bars. Data were normalized to the resazurin conversion (viable cells). Data represent an average of N = 4 independent experiments (*n* = 3). Bars represent mean ± SEM. *** *p* ≤ 0.001 as indicated.

Another important function of the liver is the synthesis of albumin [42]. As depicted in Figure 5, after 3 days of culture, twice as much albumin production by PHHs was measured in Optimaix-3D cultures compared to the same PHHs cultured in 2D. On day 10, albumin production drops in 2D cultures by approximately 38% compared to day 3. On the contrary, in Optimaix-3D cultures, albumin synthesis even increased with increasing culture time, resulting in a 16-fold higher albumin production in Optimaix-3D cultures compared to 2D cultures.

#### 3.3.3. The Activity of CYP Enzymes was Constant in 3D over 10 Days

**Figure 6:**
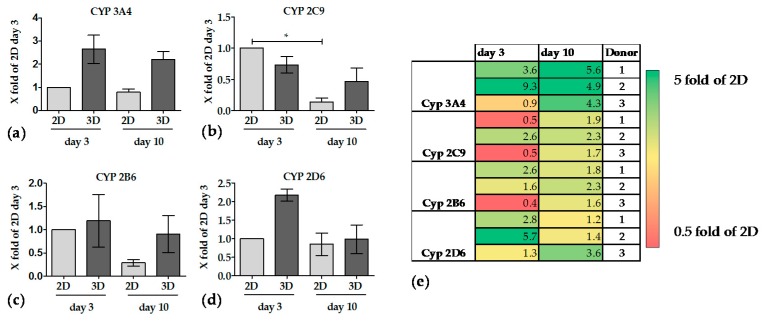
Activity of CYP enzymes, which are involved in drug metabolism. Supernatants were collected after incubation with specific substrates as indicated in Materials and Methods at days 3 and 10 of culture. (a–d) shows the CYP activities of CYP 3A4, 2C9, 2B6 and CYP 2D6. Values of 2D cultures are summarized in bright bars. Values of 3D cultures are summarized in dark bars. Bars represent mean ± SEM of three independent experiments (N = 3, *n* = 3) * *p* ≤ 0.05 as indicated. (**e**) Overview of the individual donor differences is represented in a heat map showing the CYP activities in 3D cultures compared to 2D cultures.

Basal activities of CYP3A4 and CYP2D6 in PHHs were relatively low when cultured in 2D, thus no significant drop in activity was detected over 10 days. In contrast, both enzyme activities were more than doubled when PHHs were cultured on Optimaix-3D scaffolds. CYP3A4 activity remained more or less constant over the culture period of 10 days (Figure 6a). On the contrary CYP2D6 activity showed a time-dependent decrease over 10 days in 3D culture but remained higher than in 2D when PHHs were cultured on Optimaix-3D scaffolds (Figure 6d). At day 3 in culture CYP2C9 and CYP2B6 activities were comparable between PHHs in 2D and in 3D (Optimaix-3D scaffolds). While their activity strongly declined in 2D cultures, they remained stable in 3D (Optimaix-3D scaffolds) cultures over 10 days (Figure 6b,c).

For all measured CYP activities, a high donor variance was detected, which is in agreement with the literature [43]. For a better overview, activities of each donor are also presented in a heat map in Figure 6e.

## 4. Discussion

For many years, it has been known that due to large inter-species differences in metabolism, animal models are not able to predict hepatotoxicity in humans reliably [4]. Although great efforts have been made to overcome this deficit in the past 20 years, so far no reproducible system exists to predict in vitro metabolism in humans. It is widely accepted that neither hepatic cell lines nor stem cell-derived hepatocyte-like cells are able to mimic the metabolism of drugs in vitro [15]. The only exception is the so-called ‘gold standard’ of PHHs, which show a metabolic profile comparable to the in vivo situation for a limited time. However, the use of PHHs as a routine test system for the pharmaceutical industry is limited because of several reasons.

First, the cells are not always or at any time available in sufficient (large) quantities. Additionally, they can only be isolated in specialized centers from liver capsules, which are obtained during large tumor operations. Besides, the fact that the cells’ metabolism might be affected due to the medication of the patients, the isolated cells need to be distributed to academic, regulatory affairs or industrial research institutions. Thus, PHHs need to be shipped from point A to point B. Nowadays, there are three standard methods for this procedure: (i) either the cells are cryopreserved; (ii) sent in suspension at 4 °C; or (iii) directly plated onto culture plates [15]. Each of these procedures has advantages and disadvantages. While experiments using cryopreserved PHHs can be planned ahead, the cells show a massive loss of viability associated with a reduced metabolic activity after thawing [40,41]. As also demonstrated in the present manuscript, transport of PHHs at 4 °C in suspension causes a massive loss of cell viability. Although the viability could be significantly improved by Percoll density gradient centrifugation, the overall cell numbers were strongly reduced. Furthermore, their adherence and consequently metabolic activity was affected, which is in line with our laboratory observations and other published reports [40,41]. In order to prevent this, many attempts have been made to improve cryopreservation and thawing of PHHs [15], or to optimize the solution, in which the cells are transported in cell suspension [16]. Despite all efforts that have been made in recent years, the shipping results remain unsatisfactory, with a frequently high loss of cell viability, cell attachment and function [14,44].

Another limitation is the conventional monolayer culture technique for PHHs, which is far from its natural environment and results in a reduced and limited metabolic capacity [12,45]. According to PubMed in the past 10 years over 600 papers on hepatocytes and 3D culture were published. This research led to substantial improvement in the field of bioengineering with the aim to culture PHHs in an environment that resembles the liver [18]. Nevertheless, direct comparison among the different scaffolds and systems is barely possible due to the various technologies used. Custom-made scaffolds frequently lack reproducibility. Thus, we have chosen here a scaffold, named Optimaix-3D, which is reproducibly manufactured (by Matricel) in a controlled freezing process from a defined collagen dispersion. This procedure results in an even pore structure (mean pore diameter of 88.9 μm) [46]. Studies have shown that PHHs require the interaction with Extracellular matrix (ECM) components, such as collagen, to maintain their specific functions [47]. The ECM protein collagen plays an important role in the maintenance of organs and tissues [48]. It is therefore often used as a biomaterial in medical application and in bioengineering [49]. It is biodegradable and in contrast to albumin or gelatin only weakly antigenic [48]. Collagen is also one of the important ECM proteins of the heathy liver [50]. As such, the Optimaix-3D scaffold, which is made of collagen, could potentially be an ideal carrier for PHHs for transport purposes and/or metabolic studies.

In our hands, the hydrated Optimaix-3D scaffold showed a stiffness of approximately 7.5 kPa. This stiffness is in the range of a healthy liver, which has a stiffness between 2.9 kPa and 6 kPa, depending on individual differences and the method used for measurement [23,51,52]. Compared to plastic, which has a stifness in the gigapascal range [22], the scaffold is in terms of the stiffness within the physiological range of a healthy human liver [23,53]. The stiffness of the scaffold seems to be important not only for in vitro studies [22] but also in vivo, as studies have clearly shown that liver stiffness negatively influences the drug metabolism in patients [53].

There are not many studies regarding the ideal pore size of scaffolds for the cultivation of PHHs. However, Ranucci et al. described that cell adherence as well as albumin production is particularly high at pore sizes of 10 μm and 80 μm, but not at a pore size of 18 μm. The authors explain their findings with the fact that the cell morphology seems to be best preserved in cultures with a pore size of 10 μm, while cell-cell contacts might be best formed in 80 μm pores [20]. Optimal cell-cell-interaction might be responsible for the improved functionality of the Optimaix-3D scaffold which has a pore size of 88 μm. As the authors of this study further explained, the effects of 80 μm pores is particularly high in terms of functionality, when the cell concentration is relatively high to form cell-cell contacts [20]. This is also the case in the Optimaix scaffold, on which 300,000 cells are cultured at a scaffold volume of 30 mm^3^. From physical scaffold characterization as well as microscopic analyses of cryogels, it is well known that interconnected pores allow better cell migration and deliver an optimal nutrient supply [21]. Compared with other cryogels described in the literature for the cultivation of various cell types, including liver cells e.g., Damania et al. 2018, Amirikia et al. 2017, and Galperin et al. 2010 [54,55,56], Optimaix-3D scaffolds have a very ordered and directed pore structure, which is most likely the reason for the very high permeability and high flow rates measured. Since hepatic cord structures are also composed of microscale alignments of linear unit structures [57], the Optimaix-3D scaffolds might imitate this natural environment as seen in Figure 2c.

We first tested if Optimaix-3D scaffolds could serve as an alternative approach for commonly used cryopreservation PHHs or shipping in cell suspension at 4 °C. We and others have clearly shown [15,44] that the shipment of PHHs in suspension results frequently in a significant loss in quantity and viability of cells. Although the percentage of viable cells can be largely restored by Percoll density gradient centrifugation, the damage caused by the cooling cannot be compensated, resulting in a lower cell attachment and reduced function [58,59]. It is noteworthy that PHHs shipped on scaffold survived the transport much better than PHHs plated directly onto conventional culture plates in the monolayer technology (Figure 3c) and have a better metabolic function. A comparison of different shipment methods which also include cryopreservation of the cell could be find in Table 4. On an Optimaix-3D scaffold, with a height of 3 mm, 500,000 PHHs can be seeded. This scaffold easily fits into a cavity of a 96-well-plate. In contrast, to plate the same amount of cells in 2D requires 20–25 cavities of a 96-well-plate. The seeding efficiency on the scaffold is approximately 80% compared to 2D, which is comparable to that of other scaffolds described in the literature [60].

Furthermore, it is possible that the cell amount, which can be loaded on the scaffold, can be further improved by applying a fluid flow. A study carried out by Thevenot et al., which compared various seeding methods, showed that the dynamic seeding methods allowed a higher loading capacity [61]. So far, it is extremely difficult to detach cells quantitatively from scaffolds without a substantial cell loss [62]. Therefore, the methods for cell de-attachment from scaffolds should be improved in the future.

Our data of cultured PHHs on Optimaix-3D scaffolds showed significantly improved cell viability and an increased metabolic activity of cells over a culture period of 10 days.

With regard to the urea production in 2D cultures, we observed that urea production remains largely constant between day 3 and day 10. It is noteworthy that PHHs cultured onto the Optimaix-3D showed a continuous higher basal urea formation than 2D cultures over ten days. In 2D cultures, the addition of ammonium chloride plus ornithine results in an increased urea production, on day 3 but not on day 10. However, the addition of ammonia alone is not metabolized by primary human hepatocytes. It is well known that cellular ATP is necessary for ammonia uptake into the urea cycle to form urea [63]. Therefore it is conceivable, that a high cellular loss of ATP during liver cell transportation in suspension [14] is responsible for the lack and/or delayed urea formation. In the same line of evidence Liu et al. clearly showed that cold storage of cells reduces the metabolic testing capacity, including the ammonia detoxification capacity [64]. Based on these data, it seemed that hepatocytes recover faster in 3D than in 2D cultures, suggesting a lack of additional cofactors necessary to fully transform ammonia into urea.

In the case of albumin synthesis, there is even an increase in albumin production between day 3 and day 10, when PHHs are cultured on Optimaix-3D scaffolds, while in 2D a drop in albumin production was observed. This finding supports ours and others hypotheses that the 3D environment in general and the Optimaix-3D scaffold in particularly maintains the metabolic function of PHHs [18]. An explanation for this high basal urea and albumin synthesis observed in our Optimaix-3D cultures could be the naturally occurring zonation of the liver. Cells that receive high levels of nutrients and oxygen (periportal cells) are more involved in urea detoxification and albumin production, while perivenous cells (low oxygen) are involved in drug metabolism [65]. The open pore structure of Optimaix-3D scaffolds may result in better oxygen and nutrient supply for liver cells. This effect may be dominant at the beginning of the experiment, while the cells in 2D are still in a differentiated state. In the course of the experiment, the cultivation of cells on a scaffold leads to a slowdown of de-differentiation, which would be another key advantage over 2D cell cultivation [15].

Thus, it is not surprising that the effect of the 3D cultivation on CYP activity was not as pronounced early in the culture period, when CYP activities in 3D were only slightly higher than in 2D cultures. However, over a 10-day culture period, the CYP activities remained fairly constant in the PHHs cultured on the Optimaix-3D scaffolds, while CYP activities in 2D rapidly declined. This is in line with another report, showing that a 3D environment has a positive effect on the CYP activities [18]. When we compare our results to already published data, the CYP enzyme activities in 2D confirm or previous findings. As shown by Lin et al. the RNA expression of CYP2B6 and CYP2C9 has been declined after a period of five days. In case of CYP3A4 there is almost no RNA and Protein expression after 7 days [10]. In recent years, much has happened to improve the cultivation of hepatocytes, especially the cultivation of hepatocytes in spheroids or on scaffolds show positive results. For example, the culture of rat hepatocytes on a silk scaffold showed an increase in albumin production over 10 days, however, only in co-culture with hepatic stellate cells. The urea production at later time points is also higher both in 3D and in the 2D co-culture. The metabolic activity of the CYP enzymes (CYP3A, CYP1A2) was only measured after 5 days. However, at this time point the cells in co-culture seemed to improve the metabolic function more than in the 3D system. However, the authors have not normalized these data, therefore, it cannot be ruled out that the positive effects seen in the 2D co-culture is only due to an improved survival of the hepatocytes [24]. Damania et al. have published a cryogel coated with extracellular liver matrix which should mimic the natural ECM of the liver. These authors observed an improved functionality of human hepatocytes plated on coated scaffolds, including albumin and urea production, especially on day 3. Unfortunately, a comparison with the corresponding 2D culture was not performed [54]. In the same line of evidence, Bell et al. showed a significantly improved maintenance of hepatocyte function by cultivating liver cells in a spheroid culture. Depending on the investigated CYP enzymes, maintenance of the activity was possible over 10–14 days. In this publication, liver cells in spheroids were compared with cells cultured in a Matrigel sandwich. Interestingly, Bell et al. performed their experiments in a 96-well format using ultra-low attachment plates with very low cell numbers (1500 viable cells per well), which allows the use of high-throughput methods to a certain extent. Since the formation of spheroids takes approximately 7–10 days, a direct comparison with 2D and the same primary hepatocytes is impossible, because of declining enzyme activity in 2D. Therefore, the authors have normalized their results to the amount of seeded cells [66] which does not really represent the cell number on the day of the measurement. Siltans et al. used another method to form hepatocyte spheroids: microcapsules with a liquid core and poly-(ethylene glycol) gel shell that allowed to form spheroids with only 150 cells per capsule. However, several capsules were plated per well, to obtain measurable results [5]. While the encapsulated hepatocytes showed an increased albumin production between day 4 and day 6, the corresponding 2D culture showed a decrease in albumin production. These authors claim that urea production on day 8 is highest in the encapsulated hepatocytes compared to the controls, but no comparison to other time points has been performed. In addition, the authors have executed co-cultures with murine fibroblasts, which slightly improved the metabolic function of the hepatocyte spheroid culture. The albumin production of the co-culture setup showed a continuous increase over 12 days. A direct comparison among different papers of other metabolic functions is again difficult since these authors have chosen only one particular time point [67].

In summary, many other 3D cultivation methods have been published with partly very complex compositions, which makes a controlled standardization and comparison to other models almost impossible. However, the scaffold described in this manuscript is characterized by a high degree of reproducibility. In addition, Optimaix can be applied to routine 96-well-plates that allows a high-throughput process. Other recent work with spheroid/organoid 3D cultures seems to be similar to standardize to 96-well formats; however, the normalization of results to the applied cell number and a direct comparison with 2D is difficult [68,69]. The reason is that the spheroid formation takes time, which does not allow a direct comparison with the basal PHH metabolic activities [70]. Finally, there is also evidence showing no metabolic improvement of hepatocytes cultured onto 3D spheroids compared to conventional 2D culture [71].

## 5. Conclusions

Our results clearly show that the viability of the cells can be maintained significantly better by transport on the Optimaix-3D Scaffold from Matricel compared to the conventional shipment as a cooled suspension. Additionally, it is possible to utilize this scaffold to maintain the main hepatic functions, such as drug metabolism, urea production, and albumin synthesis over a period of 10 days. Furthermore, the scaffold has nearly the same stiffness as a healthy liver. With its high porosity and permeability, it is not only ideal for supplying the cells with nutrients, but also for use within a bioreactor. Due to the good biocompatibility of the collagen used for the scaffold, it might be also possible to use a scaffold seeded with PHHs in regenerative medicine.

## Figures and Tables

**Table 1:** Substrates, concentrations, conditions, and measured reactions of the CYP activity measurement.

Substrate	Isoenzyme	Incubation Time in h	Concentration	Reaction
Bupropion	CYP 2B6	1	100 μM	Bupropion-hydroxylation
Diclofenac	CYP 2C9	1	9 μM	Diclofenac-4′-hydroxylation
Testosterone	CYP 3A4	1	50 μM	testosterone-6β-hydroxylation
Bufuralol	CYP 2D6	2	9 μM	Bufuralol-1-hydroxylation

**Table 2:** Characteristics of Optimaix-3D scaffold.

Optimaix-3D Scaffold	Pore Size (Mean Diameter/μm)	Porosity (%)	Permeability (μm^2^)	Water-Uptake (%)	Swelling-Ratio (%)
**Average**	88.9	96.3	54.5	97.1	3386.6
**Standard Deviation**	21.1	0.3	4.0	0.1	127.2

**Table 3:** Loss of living hepatocytes by shipping and subsequent purification.

	Amount of Living Cells before Shipment (in mio)	Viability before Shipment	Amount of Living Cells after Percoll Purification (in mio)	Viability after Percoll
**Donor 1**	119	80%	54	76%
**Donor 2**	111	85%	43	74%
**Donor 3**	78	83%	25	80%
**Average**	103	82%	40	77%

**Table 4:** Comparison of various shipment methods.

Shipment Method	Survival of the Cells after Shipment	Adherence Time before Shipment	Advantages	Disadvantages	Ref.
Suspension	Low	No	Cells can be plated out by the recipient on demand	Cell loss during shipment, Reduced cell attachment metabolic capacity in culture	[14,44,59]
Cryopreserved hepatocytes	Low	No	Cells can be used anytime, anywhere	Massive cell loss during freezing/thawing, Low metabolic activity	[14,40]
2D culture	High	4 h	Cells can be plated in the different well-plate formats	Cell shipment requires a large volume. Risk of contamination	[15,17]
Optimaix-3D Collagen Scaffold	High	2 h	Seeding large cell numbers in a low volume	Cell detachment difficult	This work
			Cells maintain metabolic functions over 10 days		

## Abbreviations

PHH	Primary human hepatocytes
NIH	National Institutes of Health
RGD	Arginylglycylaspartic acid
CYP	CYP450 monooxygenase
ECM	Extracellular matrix
EDC	1-ethyl-3-(3-dimethylaminopropyl)carbodiimide hydrochloride
